# RNA sequencing enables neoantigen discovery and vaccine validation in breast and lung cancer

**DOI:** 10.3389/fimmu.2025.1682312

**Published:** 2025-10-08

**Authors:** Hongye Hu, Yicheng Xiong, Danhong Lu, Weihong Sun, Xiaoping Su, Danni Mo, Lu Chen, Guan Wang, Jiayan Wang, Xiaohua Zhang, Mingdong Lu, Guanli Huang

**Affiliations:** ^1^ The First Affiliated Hospital of Wenzhou Medical University, Department of Breast Surgery, Wenzhou, China; ^2^ Alberta Institute, Wenzhou Medical University, Wenzhou, China; ^3^ The First Affiliated Hospital of Wenzhou Medical University, Department of Thyroid Surgery, Wenzhou, China; ^4^ Biotherapy Center, Qingdao Central Hospital, University of Health and Rehabilitation Sciences (Qingdao Central Hospital), Qingdao, China; ^5^ Wenzhou Medical University School of Basic Medical Sciences, Wenzhou, China; ^6^ The Second Affiliated Hospital of Wenzhou Medical University, Department of Gastrointestinal Surgery, Wenzhou, China; ^7^ Zhejiang Key Laboratory of Intelligent Cancer Biomarker Discovery and Translation, First Affiliated Hospital, Wenzhou Medical University, Wenzhou, China; ^8^ Anatomy Department, School of Basic Medical Sciences, Wenzhou Medical University, Wenzhou, China

**Keywords:** neoantigen, RNA-Seq, immunotherapy, breast cancer, lung cancer, peptides

## Abstract

**Introduction:**

Neoantigens have emerged as promising targets for personalized cancer immunotherapy due to their tumor-specific immunogenicity. However, current neoantigen prediction methods relying on combined DNA/RNA sequencing are costly and time-consuming, limiting clinical applicability. This study aimed to establish a streamlined neoantigen identification pipeline using RNA sequencing alone, evaluating its efficacy in breast and lung cancer models.

**Methods:**

We conducted neoantigen profiling of human and mice cancers using an in silico prediction pipeline based only on RNA sequencing. We also performed neoantigen-specific T responses experiments using autologous BMDCs and PBMCs with the predicted neoantigen peptides, and ultimately demonstrating significant antitumor efficacy in murine models through *in vivo* therapeutic evaluation.

**Results:**

We identified neoantigens in mice breast cancer cell 4T1, lung cancer cell LLC and one breast cancer patient based only on RNA sequencing. *In vitro* experiments demonstrated that these neoantigens triggered specific T-cell responses in BALB/c mice and the patient. Mechanistic studies revealed an increased proportion of CD3+/CD137+ T cells in the RNA-derived neoantigen peptide group, with significant infiltration of CD3+/CD137+ T cells into tumor tissues.

**Conclusion:**

RNA sequencing alone enables efficient neoantigen prediction and vaccine design, and the neoantigen vaccine can elicit an antitumor reaction against mouse breast cancer and lung cancer. The study showed that neoantigen prediction using RNA sequencing alone holds promise as a novel immunotherapeutic approach for cancer patients.

## 1 Introduction

Neoantigens, arising from various tumor-specific alterations within cancer cells, include genomic mutations, aberrant RNA splicing, post-translational modifications, and integrated virus open reading frames. These neoantigens, considered heterogeneous compared to normal tissues, are potential targets for tumor-specific immunotherapy due to their ability to trigger immune responses. Rapid identification of these neoantigens using next-generation sequencing and bioinformatics tools forms the foundation for developing cancer immunotherapies (1). Preliminary clinical research results on personalized neoantigen vaccines indicate robust tumor-specific immunogenicity and initial antitumor activity in melanoma and other cancer patients.

Recent advancements in next-generation sequencing enable fast and high-throughput prediction of neoantigens. Current standard methods for predicting neoantigens involve four main steps (2–4): 1) identifying somatic variations through Whole Exome Sequencing (WES) data from tumor and normal tissues, 2) analyzing gene expression through RNA-seq in tumor materials, 3) selecting non-synonymous mutations expressed in tumor samples, and 4) predicting the binding affinity of mutation regions with patient MHC using computational algorithms like NetMHCpan. While standard prediction methods combining whole-exome sequencing (WES) and RNA sequencing (RNA-seq) are capable of comprehensively identifying candidate neoantigens and have been successfully employed in clinical trials, significant limitations persist. Standard DNA-based approaches (e.g., WES) often fail to capture mutations occurring in non-coding regions, structural variations, or subclonal populations with low variant allele frequency (VAF < 5–10%) (5). Additionally, spatial and temporal tumor heterogeneity (6) limits the representativeness of bulk sequencing. Prohibitive costs and time-consuming analytical workflows also significantly limit access to standard prediction methods. Hence, the refinement of neoantigen identification methods is imperative.

Classical sequencing methods for neoantigen identification require three core data sets: 1) gene variation data, 2) gene expression data, and 3) MHC-peptide binding affinity data. RNA-seq precisely isolates RNA to reverse-transcribe it into DNA, utilizing high-throughput sequencing technologies, this process collects nearly all transcriptome data from a specific species, tissue, or organ under specific conditions. RNA-seq data is commonly employed for gene expression analysis, detection of gene fusions, identification of splicing events, and can be utilized for detecting somatic variations in cells (7, 8). Previous studies suggest using RNA-seq data for mutation detection is a feasible and cost-effective tool (9–13). Moreover, RNA sequencing allows MHC analysis (14). Therefore, we speculate that by utilizing RNA sequencing, it may be possible to directly analyze neoantigens, thereby significantly reducing the cost associated with neoantigen identification.

Although existing RNA-Seq-based pipelines like ASNEO, INTEGRATE-neo, and ScanNeo (15–17) have established computational frameworks for neoepitope prediction and demonstrated clinical correlations, these studies primarily rely on retrospective bioinformatic analyses without direct experimental validation of immunogenicity or therapeutic potential. Our work significantly extends these findings by providing comprehensive functional validation through both *in vitro* and *in vivo* models.

In this study, we performed RNA sequencing and neoantigen prediction by using mouse breast cancer 4T1 cells, mouse lung cancer cells (LLC) and one breast cancer patient. *In vitro* experiments demonstrated that these RNA-derived neoantigens triggered specific T-cell responses in mice and the patient. Finally, RNA-derived neoantigen showed a noteworthy improvement in number and antitumor effect compared with neoantigen based on conventional method. This suggests that using RNA sequencing alone holds promise as a novel immunotherapeutic approach for cancer patients.

## 2 Methods

### 2.1 Samples

BALB/c and C57BL/6 mice (Shanghai Jiesijie Laboratory Animal Co., LTD, Shanghai, China) were kept under specific pathogen-free conditions in accordance with the Ethics Committee of Wenzhou Medical University, Wenzhou, China. 4T1 and LLC cell line was purchased from BeNa Culture Collection (lot number: BNCC273810 and BNCC100069). The cells were cultured in RPMI1640 medium (Gibco, 31870082, Beijing, China) supplemented with 10% foetal bovine serum (FBS) (Gibco, 10099141C, Beijing, China), 100 U/mL penicillin, and 100 µg/mL streptomycin (Gibco, 10378016, Beijing, China) at 37°C and in the presence of 5% CO2.

Tumor sample from breast patient was obtained from biopsy specimens. The remainder of the tissue was immediately frozen and stored in vapor-phase liquid nitrogen.

### 2.2 DNA/RNA extraction and sequencing

RNA from 4T1 and LLC cells and tumor sample were extracted in triplicate with the aid of the RNA extraction agent (InvitrogenTM, 12183555, Shanghai, China). Genomic DNA from 4T1, LLC cells and tumor sample were extracted in triplicate with the aid of the DNA extraction agent (Thermo Scientific™, K0512, Shanghai, China). Using 2μg of total RNA, barcoded mRNA-seq cDNA libraries were constructed with Illumina TruSeq RNA Prep Kit v2. The DNA was were constructed with Agilent Sure Select Human All Exon 44- Mb version 2.0 bait set. All libraries were sequenced on an Illumina NovaSeq6000 for 50 cycles at Mingma Technologies (Shanghai, China). Only high-quality reads that passed the quality filters were preserved for the sequence analysis. The resulting RNA sequence was submitted to the National Center for Biotechnology Information (NCBI) GenBank under BioProject number SAMN31572059.

### 2.3 Data processing and neoantigen prediction

#### 2.3.1 Neoantigen predicted by RNA sequencing

Quality control and preprocessing of FASTQ files were performed using fastp v0.16.0 followed by mutation analysis based on Sentieon Genomics software. For the mutation analysis, the first step was to map reads contained in the FASTQ files to a reference genome contained in the FASTA file. In the second step, the same RNA molecules sequenced several times were removed and the RNA reads were split into exon segments. Then the quality scores assigned to individual read bases of the sequence read data were modified to remove experimental biases caused by the sequencing methodology. And finally, the sites where the data displays variation relative to the reference genome were identified, and the genotypes for each sample at that site were calculated. The measure of RPKM/TPM were processed with hisat2 (v2.1.0) and stringtie (v1.3.4d) in paired-end RNA-seq experiments. Filtering thresholds for RNA-seq variant calling include Depth of Coverage (DP, ≥50× for tumor tissue, ≥30× for normal tissue), Allele Depth (AD, ≥5× for tumor tissue, ≥3× for normal tissue), base quality score cutoffs (≥Q20), strand bias filtering (SOR ≤ 5.0), VAF (≥5% for tumor somatic cells) and TPM (≥1). We used all HLA class I binding algorithms implemented in seq2HLA (v2.3) to predict MHC class I (A, B, C) and class II (DQA1, DQB1, DRB1, DRA, DPA1, DPB1) in breast cancer patients. Peptide screening was performed using MHC molecules strictly matched to the genetic background of the experimental animals: C57BL/6 mice (H-2b haplotype): Screens utilized endogenous MHC molecules, including H2-Kb (class I), H2-Db (class I), and H2-IAb (class II). BALB/c mice (H-2d haplotype): Screens employed endogenous MHC molecules, including H2-Kd (class I), H2-Dd (class I), and H2-IAd (class II). The IC50 binding score of epitopes were calculated using NetMHCpan and NetMHC-IIPAN software. In the selection of neoantigen epitopes, we got the priority score by considering multiple weighted dimensions, including but not limited to: MHC binding affinity differential between mutant and wild-type peptides (e.g., ΔIC50 or ΔRank), gene/transcript expression levels (e.g., RPKM/TPM), variant allele frequency (VAF), mutation concordance between DNA and RNA (supporting transcribed variants), antigen presentation probability and immunogenicity predictions. The weighting scheme is optimized based on training datasets or clinical validation.

#### 2.3.2 Neoantigen predicted by conventional approach

In addition, we performed neoantigen prediction with a conventional approach (18). Conventional neoantigen profiling were performed according to previous study (19). DNA reads were using BWA compared with the reference genome mm10 for mouse mutation detection or UCSC human reference genome hg19 (GRCh37) for breast patient (20). Somatic mutations were detected with Mutect2 and Somaticsniper and confirmed by DeepSNV (21). Indels were detected by Metect2 and Strelka and then manually confirmed by Integrative Genomics Viewer at both DNA and RNA levels.

For RNA-seq, the qualified reads were aligned to human genome reference hg19 with GENCODE gene annotation using STAR in breast patient (22). And RNA reads were compared with the mm10 reference genome and transcriptome using Bowtie in mice (23). Then TPM were calculated to quantify the expression levels for each gene. For each nonsynonymous mutation, all possible mutated peptides containing mutated amino acids with length ranging from 8 to 11 mer were extracted. The binding affinity to corresponding MHC molecules was predicted by NetMHCpan V.4.0. Screens utilized endogenous MHC molecules, including class I (A,B,C) and class II (DQA1, DQB1, DRB1, DRA, DPA1, DPB1) in breast cancer patients, H2-Kb (class I), H2-Db (class I), H2-IAb (class II) in C57BL/6 mice (H-2b haplotype) and H2-Kd (class I), H2-Dd (class I), and H2-IAd (class II) in BALB/c mice (H-2d haplotype). Peptides with IC50<500nM were predicted to be candidate epitopes.

### 2.4 Synthetic peptides and adjuvants

The neoantigen peptides of 27 or 31 amino acids in length were synthesized by GenScript (Nanjing, China) with strict quality control (>95% purity). Polyinosinic: polycytidylic acid (poly (I:C); P9582-5MG, Sigma-Aldrich, MO, USA) was applied as subcutaneously injected adjuvant.

### 2.5 Immunization of mice

Age-matched female BALB/c and C57BL/6 mice were injected with 100 μg peptide and 50 μg poly (I:C) formulated in PBS (200 mL total volume) subcutaneously into the lateral flank. Every group was immunized on day 0, day 7 and day 14 with 1 peptide per flank. Mice were humanely sacrificed 19 days after the initial injection and splenocytes isolated using Dynabeads Mouse T Cells Kit (Invitrogen, 11413D, Shanghai, China) for immunologic testing. The bone marrow-derived dendritic cells (BMDCs) were harnessed according to a forementioned described previously (24). BMDCs were suspended in RPMI1640 medium supplemented with 10% FBS, 20ng/mL GM-CSF (BD Pharmingen, 564747). The immature BMDCs (non-adherent cells) were collected for experiments on day 6 subsequently.

### 2.6 Enzyme-linked immunospot assay

For BALB/c and C57BL/6 mice, 1×10^5^ immature BMDCs were stimulated with peptide (4 µg per peptide) for 48 h at 37 °C with 5% CO2. The culture medium consisted of RPMI1640 medium, 10% FBS, 10 ng/mL GM-CSF, 5 ng/mL IL-4 (CK15, novoprotein, Suzhou, China) and 20 ng/mL TNF (C008, novoprotein, Suzhou, China). Then, 2×10^5^ spleen cells after thawing for 24h were cocultured with the peptide-simulated BMDCs cells (5×10^4^) at 37°C for another 36 h. The specific T cell responses to each peptide were measured through an ELISpot assay using IFN-γ ELISpot plus kit (3420-2AST-10, Mabtech Limited, Swedish), according to the manufacturer’s instructions.

For patient with breast cancer, the blood samples were obtained from the patients for the isolation of PBMCs by centrifugation using a Ficoll density gradient (17544202, GE, USA), and the obtained PBMCs were suspended in KBM581 (88-581-CM, Corning, USA). 1 × 10^5^ PBMCs were pulsed with peptide (25 µM) in 200mL of culture medium respectively. The culture medium consisted of KBM581 medium, 10% FBS, and 100 U/ml IL-2 (C013, novoprotein, Suzhou, China). Half of the culture medium was exchanged with fresh medium containing the peptide (25 µM) and 100 U/mL IL-2 every three days for peptide stimulation. The IFN-γ secreting cells was determined by ELISPOT kit (3420-2AST-2, Mabtech Limited, Swedish).

Each single neoantigen peptide was tested in comparison with the positive control (CD3 as the stimulators) and negative control (splenocytes isolated or PBMCs without pulsed with peptide).

### 2.7 Antitumor effects of neoantigen vaccine *in vivo*

1×10^6^ 4T1 or LLC cells were inoculated subcutaneously into the flanks of BALB/c or C57BL/6 mice for tumor vaccination. Therapeutic immunization with the peptide vaccine was administered subcutaneously on days 1, 8 and 15 after tumor injection. The mice were divided into RNA neoantigen vaccine group (200 µg per peptide based on RNA and 50µg poly (I:C) formulated in 200 µL PBS), conventional neoantigen vaccine group (200 µg peptide based on routine approach and 50µg poly (I:C) formulated in 200 µL PBS), poly (I:C) group (50 µg poly (I:C) formulated in 200 µL PBS), and normal group (200 µL PBS) with cohort sizes (n=5 mice per group) and randomization protocol (block randomization by tumor volume).

The tumor sizes were monitored every 3 days, and mice were humanely sacrificed when tumor diameter reached 15 mm.

### 2.8 Immunofluorescence analysis and histopathological evaluation

Excised LLC mouse tumor tissues were fixed in 3.5% formaldehyde. Subsequently, immunofluorescence analysis was conducted by the Pathological Research Institute of Wuhan Servicebio Technology Co., Ltd. Microscopic analysis of IF images was performed using DS-U3 and ECLIPSE C1 Ortho-Fluorescent microscopes (Nikon, Japan).

### 2.9 Statistical analysis

GraphPad Prism 8.0 (GraphPad Software) was used for all statistical analyses. The data samples were compared using two-tailed Student’s t test, and a P value less than 0.05 were considered statistically significant.

## 3 Results

### 3.1 Neoantigens based on RNA-seq were predicted and identified from mouse tumor cells

**Figure 1:**
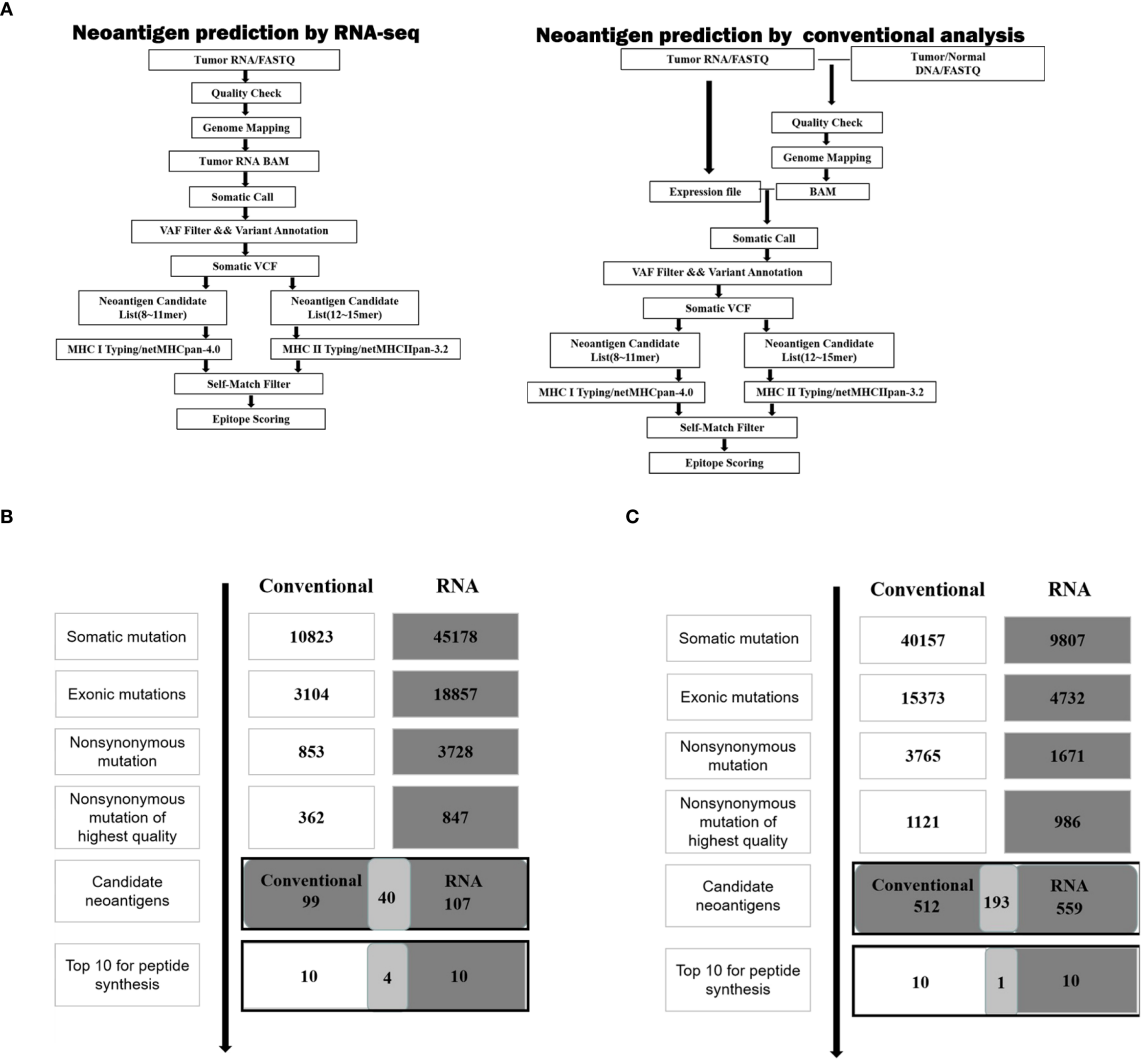
Neoantigen screening workflow and outcome in two cancer cell lines. **(A)** Schematic of the workflow described in the analysis of neoantigens. **(B)** The number of mutations obtained during neoantigen analysis in 4T1 cells. **(C)** The number of mutations obtained during neoantigen analysis in LLC cells.

According to the scheme of the study, as shown in **Figure 1A**, neoantigen prediction based on RNA-seq and conventional analysis were carried out on 4T1 cells and LLC cells.

For 4T1 cells, the mutation filtering results are illustrated in **Figure 1B**. The RNA-derived neoantigen prediction obtained more mutations compared with conventional analysis. 107 and 99 candidate neoantigens were eventually obtained by RNA-seq and conventional analysis respectively, 40 of the neoantigens were overlapped. For LLC cells, the mutation filtering results are illustrated in **Figure 1C**. 559 and 512 candidate neoantigens were eventually obtained by RNA-seq and conventional analysis respectively, 193 neoantigens were overlapped. 10 mutations with the highest comprehensive score in both groups were selected for the following research (**Tables 1**–**4**). Four neoantigens were overlapped (bold) in 4T1 group and1 neoantigen were overlapped (bold) in LLC group. The neoantigen chromosome distribution for 4T1 cells and LLC cells are shown in **Supplementary Figures S3**, **S4** and **Supplementary Figure S1B**, respectively.

**Table 1:** RNA-derived neoantigens in 4T1 cells.

Neoantigen ID	HLA allele	Allele frequency	Amino acid change	Gene symbol	Peptide position	Mut_ MHC_ ic50	Priority score	Augmented peptide
M1	H-2-Kd	0.836	H/Y	H2	2	461	83	VVVPLGKEQNYTCHVYHEGLPEPLTLRWEPP
M2	H-2-Kd	0.75	T/M	Atp10a	9	6482.9	72	GWRRPRRRRWEGRTRMVRSNLLPPLGTEDST
M3	H-2-Kd	0.8	Q/R	Rpl9	1	4030.4	68	CSHVQNMIKGVTLGFRYKMRSVYAHFPINVV
M4	H-2-Kd	0.8	S/F	Lyst	2	4081.7	64	AVLDVDGLDIQQELPFLSVGPSLHKQQASSD
M5	H-2-Kd	0.575	K/T	Zfr	7	4033.6	56	AYAAHIRGAKHQKVVTLHTKLGKPIPSTEPN
M6 (Failing synthesis)	H-2-Kd	0.558	R/W	Icmt	2	9856.8	55	MAGCAAWVPPGSEARLSLATFLLGASVLALP
M7	H-2-Dd	0.545	C/G	Scrn1	2	13181.7	53	AWLWGAEMGANEHGVGIANEAINAREPAAET
M8 (Failing synthesis)	H2-Ld	0.56	R/P	Lancl3	2	9614.6	52	LQMLLSYQEHLKPSDPELVWQSVDFLMEQEQ
M9	H-2-Kd	0.5	E/V	Eppk1	3	3266.8	49	GKATMEVKRGHLRGHVVPVWDILTSNYVSRD
M10	H2-Ld	0.909	T/I	Gm10093	4	9832.1	45	NYPLRDGIDDESYEAIFKPVMSKVMEMFQPS

**Table 2:** Conventional neoantigens in 4T1 cells.

Neoantigen ID	HLA allele	Allele frequency	Amino acid change	Gene symbol	Peptide position	Mut_ MHC _ic50	Priority score	Augmented peptide
C1	H-2-Dd	1	P/L	Dtx2	8	1654.6	100	CLSRAPRPTGPPASRLASKSHSSVKRLRKMS
C2	H2-Ld	1	E/G	Sh3d21	6	12906.2	98	IPATEDTTLDKAGTPGSTLSGNKPAKDEALD
C3(M6)	H-2-Kd	0.778	R/W	Icmt	2	9856.8	77	MAGCAAWVPPGSEARLSLATFLLGASVLALP
C4	H2-Ld	1	R/L	Dhx58	9	7777.9	75	LLETPRGKIQAKKWSLVPFSIPVFDILQDCT
C5	H-2-Kd	0.738	M/I	Polr2a	9	8697.4	56	VGALAAQSLGEPATQITLNTFHYAGVSAKNV
C6(M7)	H-2-Dd	0.563	C/G	Scrn1	2	13181.7	55	AWLWGAEMGANEHGVGIANEAINAREPAAET
C7(M8)	H2-Ld	0.581	R/P	Lancl3	2	9614.6	54	LQMLLSYQEHLKPSDPELVWQSVDFLMEQEQ
C8(M5)	H-2-Kd	0.502	K/T	Zfr	7	4033.6	49	AYAAHIRGAKHQKVVTLHTKLGKPIPSTEPN
C9	H-2-Kd	0.465	H/Y	Wdr33	2	760.3	46	MATEIGSPPRFFYMPRFQHQAPRQLFYKRPD
C10 (Failing synthesis)	H2-Ld	0.562	D/G	Chd2	6	8635.9	43	RKPRVKKENKAPRLKGEHGLEPASPRHSDNP

**Table 3:** RNA-derived neoantigens in LLC cells.

Neoantigen ID	HLA allele	Allele frequency	Amino acid change	Gene symbol	Peptide position	Mut_ MHC_ ic50	Priority score	Augmented peptide
LR1	H-2-Kb	0.996	V/A	Ndufs6	2	15.6	36	AALTFRRLLTLPRAARGFGVQVSPSGE
LR2	H-2-Kb	0.725	P/S	Plekho1	2	121.4	19	SLSRPWEKPDKGASYTPQALKKFPSTE
LR3 (LC3, Failing synthesis)	H-2-Kb	1	T/M	Emc1	2	471.3	17	LARDEFNLQKMMVMVTASGKLFGIESS
LR4	H-2-Kb	0.552	G/V	Tmem101	1	52.6	17	ALQLAISTYTAYIVGYVHYGDWLKVRM
LR5 (Failing synthesis)	H-2-Kb	0.711	E/K	Asap1	1	137.3	16	SHHLSLDRTNIPPKTFQKSSQLTELPQ
LR6	H-2-Kb	0.622	H/L	Zscan21	8	26.5	14	FSHSSNLTLHYRTLLVDRPYDCKCGKA
LR7	H-2-Kb	0.653	D/Y	Riok1	5	4.5	12	YLQVIQYMRKMYQYARLVHADLSEFNM
LR8 (Failing synthesis)	H-2-Kb	1	R/L	Extl1	9	40.4	12	VDFAFVVWQSFPELMVGFLSGSHFWDE
LR9	H-2-Kb	0.45	K/T	Nckap1	2	73.3	11	AVSHAGSMHRERRTFLRSALKELATVL
LR10	H-2-Kb	1	L/M	Plin2	1	280.2	9	MNSGVDNAITKSEMLVDQYFPLTQEEL

**Table 4:** Conventional neoantigens in LLC cells.

Neoantigen ID	HLAallele	Allele frequency	Amino acid change	Gene symbol	Peptide position	Mut_ MHC _ic50	Priority score	Augmented peptide
LC1	H-2-Db	1	S/L	Smtn	9	129.6	100	VEAPVSSEPLPHPLEAPSPEPPMSPVP
LC2	H-2-Kb	0.996	P/S	Eya3	1	16.6	100	AHILSVPVSETTYSGQTQYQTLQQSQP
LC3 (LR3, Failing synthesis)	H-2-Db	1	T/M	Emc1	9	121.4	100	LARDEFNLQKMMVMVTASGKLFGIESS
LC4	H-2-Kb	1	D/G	Leprot	7	280.2	99	VSAFGLPVVLARVGVIKWGACGLVLAG
LC5(Failing synthesis)	H-2-Db	1	P/S	Zmym1	2	116.1	99	ACSSSYNSAVMESSSVNVSMVHSSSKE
LC6	H-2-IAb	1	T/A	C77080	14	73.3	89	VLAAPAVAPGQVSAIDTSPASPSMPQT
LC7	H-2-IAb	1	T/A	Ewsr1	2	79.2	89	QAYSQPVQGYGTGAYDSTTATVTTTQA
LC8	H-2-IAb	1	Y/S	Arid1a	6	84.2	88	QRTLLDPGRFTKVSSPAHTEEEEEEHL
LC9(Failing synthesis)	H-2-IAb	1	N/S	Gale	1	24.5	70	IQLLEIMRAHGVKSLVFSSSATVYGNP
LC10	H-2-IAb	0.723	E/K	Tpd52	3	406.5	64	KVGGAKPAGGDFGKVLNSTANATSTMT

The results demonstrate that adequate amount of neoantigens could be also obtained only through RNA-seq.

### 3.2 Specific T responses can be induced *in vitro* from mice by neoantigen from RNA-seq

To assess the specific T responses of the neoantigen peptides from 4T1 cells and LLC cells by RNA-seq, 10 mutations with the highest comprehensive score based on RNA or conventional analysis were selected in both groups.

For 4T1 cells, 8 mutations based on RNA and 9 mutations based on conventional analysis were successfully synthesized for subsequent antitumor research (**Tables 1**, **2**). For LLC cells, 7 mutations based on RNA and 7 mutations derived from conventional analysis were successfully synthesized for subsequent antitumor research (**Tables 3**, **4**).

**Figure 2:**
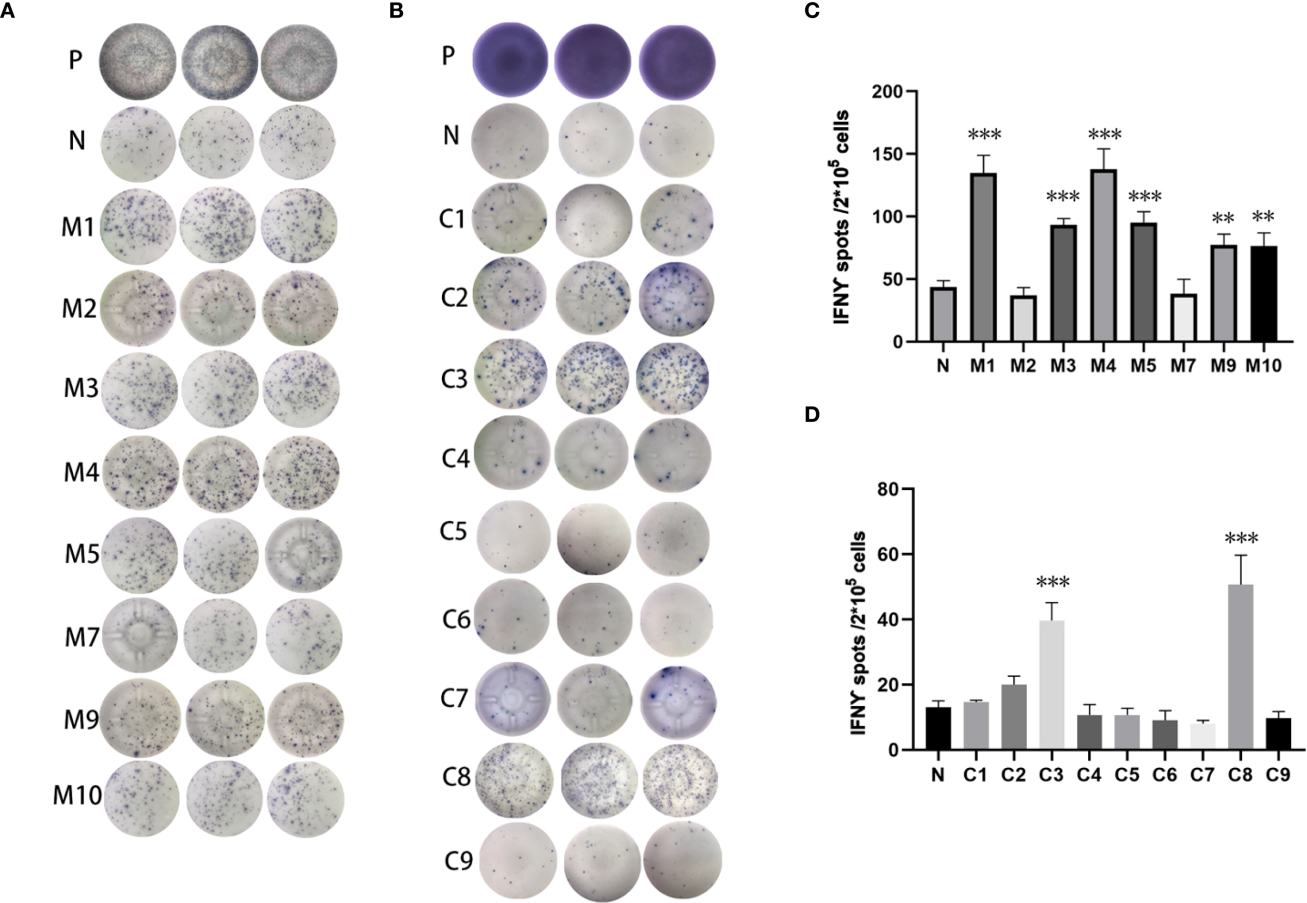
Immunogenicity assessment of neoantigen peptides from 4T1 cells. **(A)** The ELISpot assay spot images of RNA-derived neoantigen peptides. P: Positive control (CD3 stimulation), N: Negative control (unpulsed splenocytes/PBMCs) **(B)** The ELISpot assay spot images of conventional neoantigen peptides. P: Positive control (CD3 stimulation), N: Negative control (unpulsed splenocytes/PBMCs) **(C)** Statistical results of the number of spots detected in the ELISpot assay of RNA-derived neoantigen peptides. **(D)** Statistical results of the number of spots detected in the ELISpot assay of conventional neoantigen peptides. The data are presented as the means ± s.e.m. from three independent experiments. ***P<0.001, **P<0.01 were obtained for the comparison of IFN-γ production by T cells unstimulated with a peptide.

**Figure 3:**
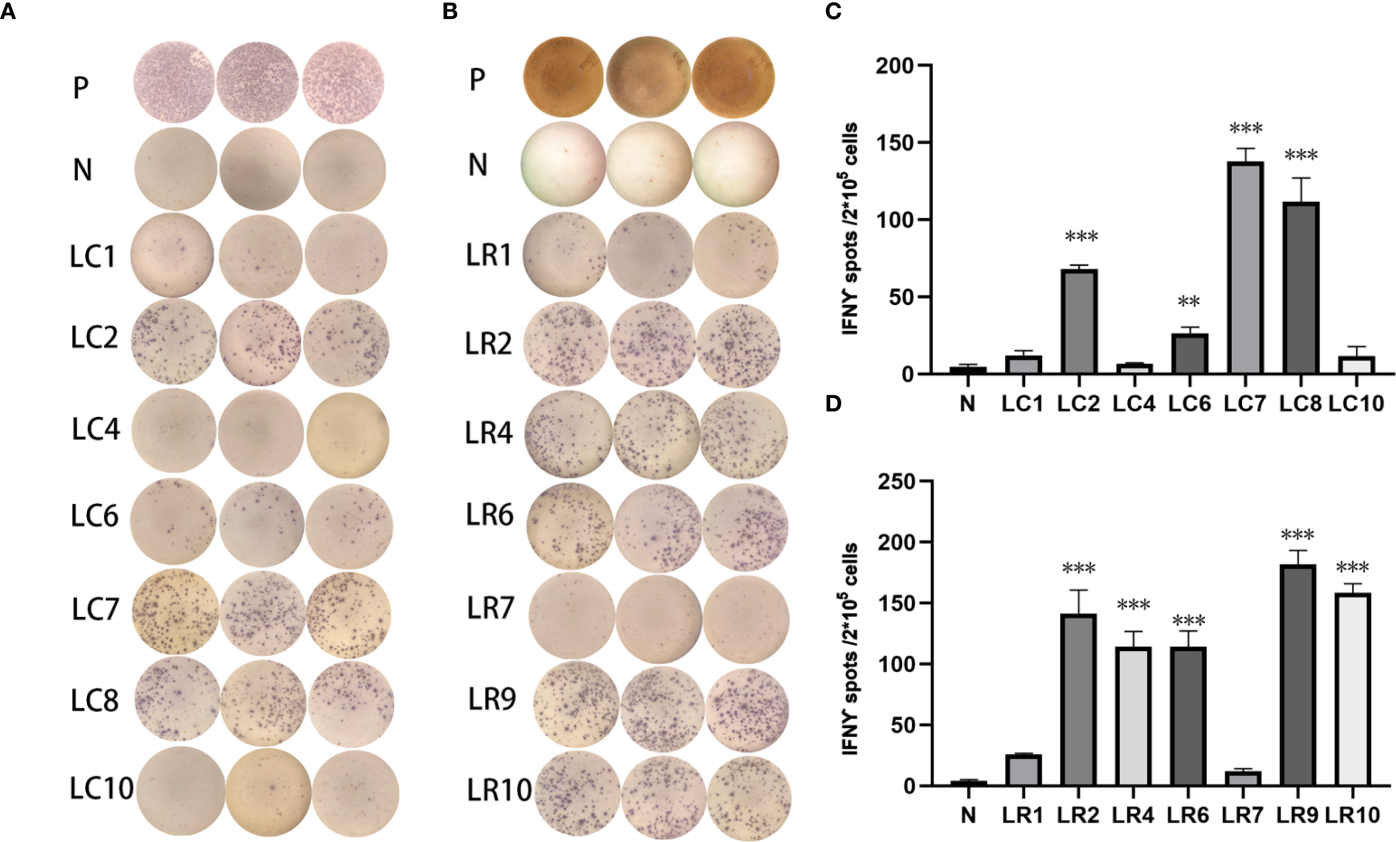
Immunogenicity assessment of neoantigen peptides form LLC cells. **(A)** The ELISpot assay spot images of conventional neoantigen peptides. P: Positive control (CD3 stimulation), N: Negative control (unpulsed splenocytes/PBMCs) **(B)** The ELISpot assay spot images of RNA-derived neoantigen peptides. P: Positive control (CD3 stimulation), N: Negative control (unpulsed splenocytes/PBMCs) **(C)** Statistical results of the number of spots detected in the ELISpot assay of conventional neoantigen peptides. **(D)** Statistical results of the number of spots detected in the ELISpot assay of RNA-derived neoantigen peptides. The data are presented as the means ± s.e.m. from three independent experiments. ***P<0.001, **P<0.01, were obtained for the comparison of IFN-γ production by T cells unstimulated with a peptide.

Three subcutaneous immunizations were performed on BALB/c mice or C57BL/6 mice using those peptides separately. Splenocytes were extracted subsequently and subjected to ELISpot experiments to evaluate the IFN-γ secretion. In 4T1 group, the results indicated that six of the RNA-derived neoantigens, namely M1 (H2), M3 (Rpl9), M4 (Lyst), M5 (Zfr), M9 (Eppk1) and M10 (Gm10093) induced significant neoantigen-specific T responses (**Figures 2A, C**), while conventional neoantigens peptides namely C2 (Sh3d21), C3 (Icmt) and C8 (Zfr) induced significant immune responses (**Figures 2B, D**). In LLC group, the results indicated that 4/7 conventional neoantigens peptides namely LC2 (Eya3), LC6 (C77080), LC7 (Ewsr1) and LC8 (Arid1a) induced significant immune responses (**Figures 3A, C**). Meanwhile, compared with conventional neoantigens peptides, 5/7 of the RNA-derived neoantigens, namely LR2 (Plekho1), LR4 (Tmem101), LR6 (Zscan21), LR9 (Nckap1) and LR10 (Plin2) induced significant neoantigen-specific T responses (**Figures 3B, D**).

The results indicated that epitope prediction strategies can identify and prioritise candidate neoantigens using RNA sequencing alone. The positive peptides were used for subsequent antitumor experiments *in vivo*.

### 3.3 RNA-derived neoantigen peptides vaccine slows down mouse tumor progression

**Figure 4:**
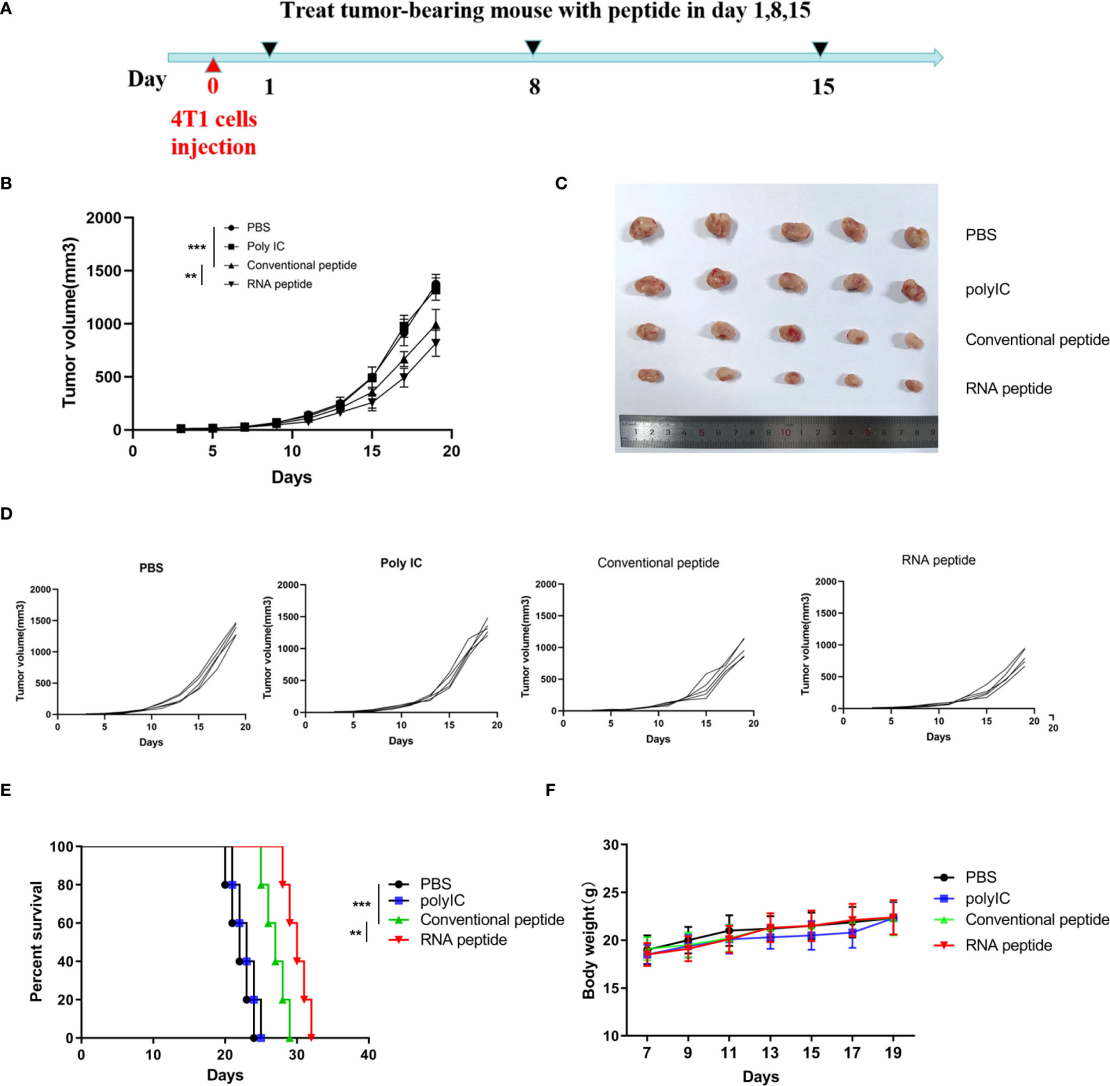
Subcutaneous 4T1 tumor model establishment, treatment, and tumor volume tracking. **(A)** The tumor volume analysis of 4T1 tumor bearing BALB/c mice at the 19th day after treatment with various formulations. The tumor bearing mice were treated with PBS (PBS), poly (I:C) alone (Poly IC), conventional neoantigen peptide (Conventional peptide, CP) and the RNA-derived neoantigen peptide (RNA peptide, RP). Treatment began on Day 1, with injections administered on Days 1, 8, and 15 to the four groups of mice. Starting from Day 0, tumor growth was measured every three days (Day 3, 5, 7, 9, 11, 13, 15, 17, 19) using calipers. **(B)** The growth curves and statistically analysis of average tumor volumes for all four groups. ****P<0.0001, ***P<0.001, **P<0.01, and *P<0.05 were obtained for the comparison of the average tumor volumes in different groups. **(C)** The shape of tumor mass removed after 19 days in different groups. **(D)** The growth curves of tumor volumes of each mouse for all four groups. **(E)** Cumulative survival of animals with 4T1 tumors in each group. **(F)** Changes in body weight analysis of 4T1 tumor bearing BALB/c mice.

To further investigate whether neoantigen peptides can serve as potent vaccines against tumor growth *in vivo*, we established the 4T1 cells induced BALB/c mouse model and LLC cells induced C57BL/6 mouse model, accomplishing subcutaneous injection according to **Figure 4A**.

We designed four treatment groups: PBS group (PBS), poly (I:C) alone group (Poly IC), conventional neoantigen peptide group (Conventional peptide), and the RNA-derived neoantigen peptide group (RNA peptide).

The mice were euthanized on Day 19 in 4T1 cells induced BALB/c mouse model. Tumor volume was quantified in **Figure 4B** and complied with the tread of **Figure 4C**. As shown in **Figures 4B–D**, tumor-bearing mice treated with neoantigen peptides, especially RNA-derived neoantigen, exhibited delayed tumor growth, whereas the injection of poly (I:C) and PBS did not restrict tumor growth. Cumulative survival curves in **Figure 4E** followed the similar trend with tumor volume. The survival time of groups containing with neoantigen peptides was significantly extended. BALB/c mice of RNA peptide group died within 32 days. Body weight for all groups gradually increased, as was shown in **Figure 4F**.

**Figure 5:**
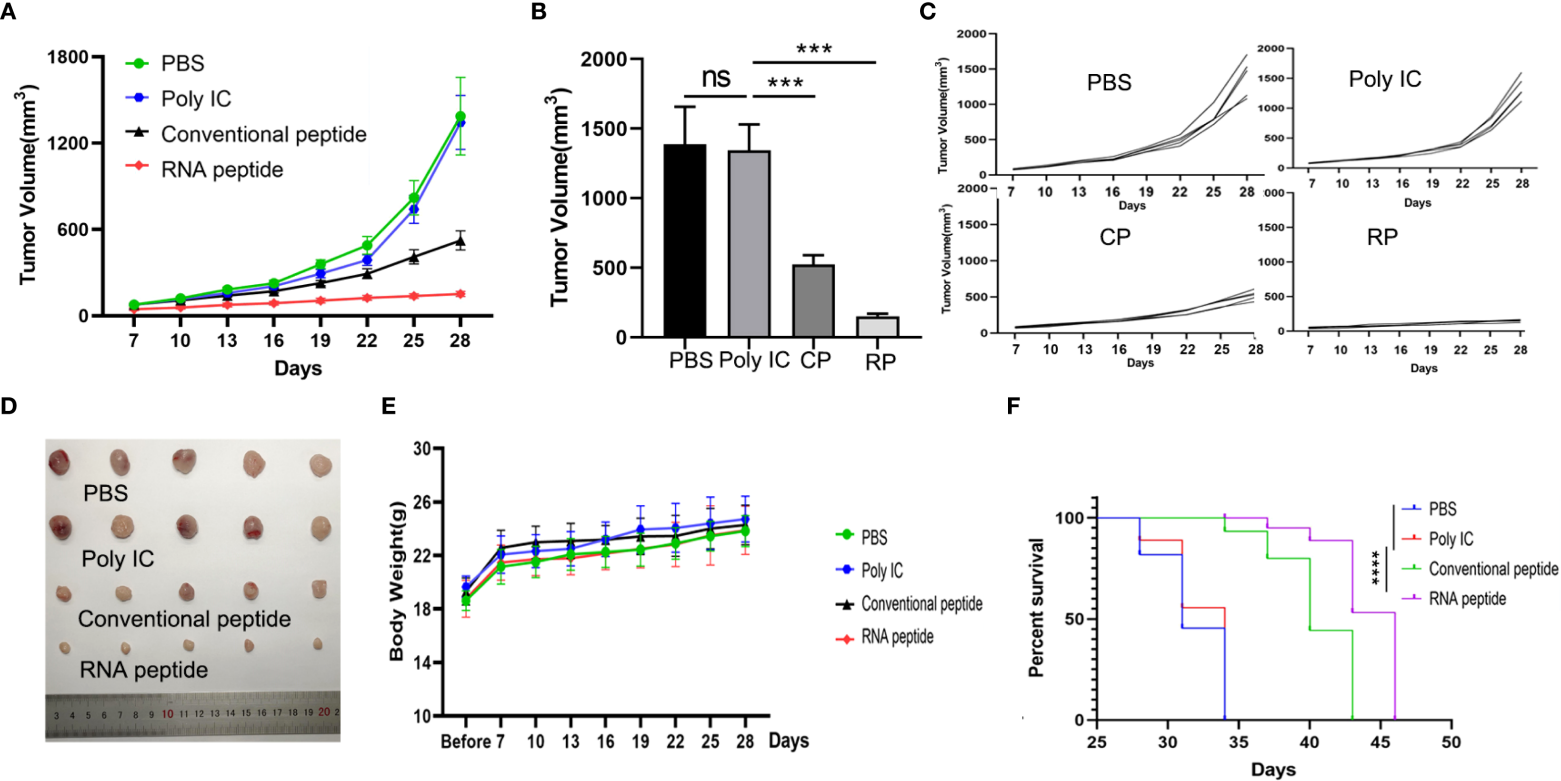
Subcutaneous LLC Tumor Model Establishment, Treatment, and Tumor Volume Tracking. **(A)** The tumor volume analysis of LLC tumor bearing C57BL/6 mice at the 28th day after treatment with various formulations. The tumor bearing mice were treated with PBS (PBS), poly (I:C) alone (Poly IC), conventional neoantigen peptide (Conventional peptide, CP) and the RNA-derived neoantigen peptide (RNA peptide, RP). The treatment schedule is the same as that of the 4T1 group. Starting from Day 7, tumor growth was measured every three days (Day 7, 10, 13, 16, 19, 22, 25, 28) using calipers. **(B)** Tumor volume measured at the 28th day. ***P<0.001. **(C)** The growth curves of tumor volumes of each mouse for all four groups. **(D)** The shape of tumor mass removed after 28 days in different groups. **(E)** Changes in body weight analysis of LLC tumor bearing C57BL/6 mice. **(F)** Cumulative survival of animals with LLC tumors in each group.

For LLC cells induced C57BL/6 mouse model, tumor volume was quantified in **Figures 5A–C**. The shape of tumor mass removed after 28 days in different groups was showed in **Figure 5B**. Tumor volume of PBS and Poly IC group was the largest, while tumor growth of Conventional peptide group was slowed down significantly, and RNA peptide group presented the smallest tumor volume. The shape of tumor mass removed after 28 days in different groups was shown in **Figure 5D**. Body weight for all groups gradually increased, as was shown in **Figure 5E**. Cumulative survival curves in **Figure 5F** followed the similar trend with tumor volume. C57BL/6 injected with PBS and poly (I:C) died within 34 days, and survival time of groups containing with neoantigen peptides was significantly extended. C57BL/6 mice of RNA peptide group died within 46 days.

In order to ascertain the presence of tumor-specific T lymphocytes in the tumor microenvironment post-treatment, we conducted immunofluorescence analysis on tumor tissues. In comparison to the other two groups, the peptide treatment groups have increased tumor tissue infiltration of T cells (CD3+CD137+ T cells) as shown in **Supplementary Figure S2**. Tumor tissues of RNA peptide group exhibited most infiltration of T cells (CD3+CD137+ T cells).

The results indicated that neoantigen peptides were safe into the body. The neoantigen peptide vaccines demonstrate a better antitumor effect than poly (I:C) therapy, especially for RNA-derived neoantigens. The strong antitumor effect of RNA peptide indicated that neoantigen prediction using RNA sequencing alone holds promise as a novel immunotherapeutic approach.

### 3.4 Immunogenic neoantigens based on RNA-seq were predicted and identified from patient with breast cancer

In order to verify whether the RNA-based neoantigen prediction is also applicable to cancer patients, we collected specimen from one breast cancer patient and conducted RNA extraction, neoantigen prediction and assessment of immunogenicity.

A 51-year-old woman was diagnosed with primary breast cancer in 2017, which had disseminated to the liver in 2019 and to lymph nodes in 2020. She had undergone chemotherapy, radiotherapy, endocrine therapy and immunotherapy treatment. We identified epitope peptides which are presented to HLA-A02:07, HLA-B15:27, HLA-B46:01, HLA-C01:02, DRB1_0901, DRB1_1501, HLA-DPA10202-DPB10202, HLA-DQA10102-DQB10303 and HLA-DQA10303-DQB10602.

**Figure 6:**
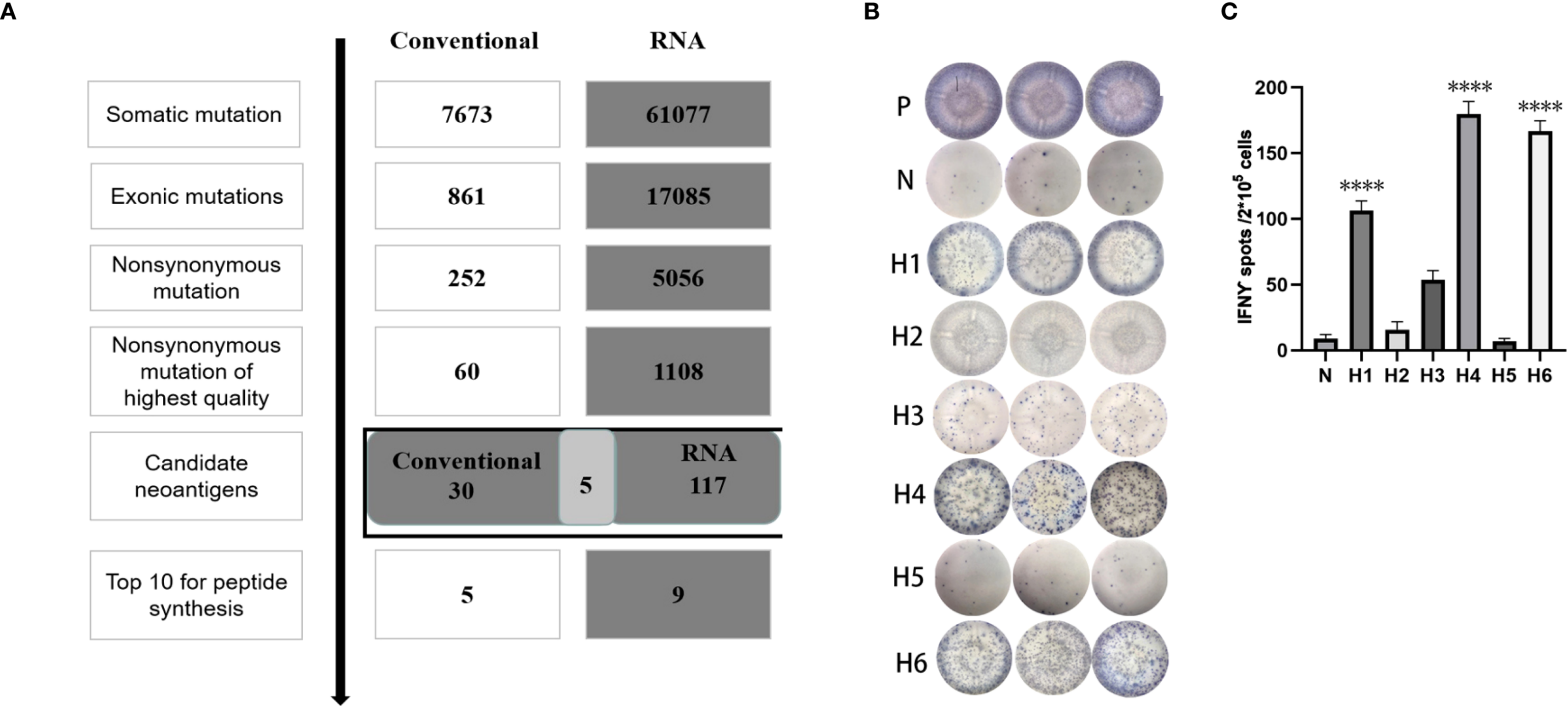
Mutation profile and immunogenicity analysis of RNA-derived neoantigen peptides in breast cancer patient. **(A)** The number of mutations obtained during neoantigen analysis in breast cancer patient. **(B)** The ELISpot assay spot images of RNA-derived neoantigen peptides in breast cancer patient. P: Positive control (CD3 stimulation), N: Negative control (unpulsed splenocytes/PBMCs) **(C)** Statistical results of the number of spots detected in the ELISpot assay of RNA-derived neoantigen peptides in breast cancer patient. The data are presented as the means ± s.e.m. from three independent experiments. ****P<0.0001, ***P<0.001, **P<0.01, and *P<0.05 were obtained for the comparison of IFN-γ production by PBMCs unstimulated with a peptide.

During the neoantigen analysis, the mutation filtering results are illustrated in **Figure 6A**. Finally, the neoantigen chromosome distribution is shown in **Supplementary Figure S1A**. 9 mutation-associated long peptides with the highest comprehensive score were selected and produced and 6 were successfully synthesized (**Table 5**). In order to identify the neoantigen peptides from patient with breast cancer with high immunogenicity, an established immunogenicity assay (25) was applied to test the immunogenicity of the synthesized neoantigen peptides (26). The results indicated that 3 of the RNA-derived neoantigens, namely H1 (DEPDC1), H4 (WDR7) and H6 (TGIF2) induced significant neoantigen-specific T responses (**Figures 6B, C**).

**Table 5:** RNA-derived neoantigens in breast cancer patient.

Neoantigen ID	HLA allele	Amino acid change	Allele frequency	Peptide position	Gene symbol	Mut_ MHC_ ic50	Priority score	Augmented peptide
H1	DRB1_1501	E/K	0.667	10	DEPDC1	180.15	39	SFKSTECLLLSLLHRKKNKEESDSTERLQIS
H2	HLA-DPA10202-DPB10202	L/P	0.646	14	PSPH	51.57	32	GFGGNVIRQQVKDNAKWYITDFVELLGEPEE
H3	DRB1_1501	I/M	0.833333	10	ZNF587B	159.69	13	HQRFGRPRWVDHKDRKEFKTSLGNMVKSCLF
H4	HLA-B15:27	G/E	0.181818	7	WDR7	991.1	9	LLCSGPSENGQTWTGEDFVSSDKVIIWTENG
H5	HLA-B15:27	K/E	0.0512821	5	SYNC	1471.4	3	ERQRQLRNGVQLQQQENKEMEQLRLSLAEEL
H6	DRB1_1501	Q/R	0.0327869	14	TGIF2	63.76	2	RDWLYLHRYNAYPSEREKLSLSGQTNLSVLQ
H7	HLA-A02:07	N/D	0.0363636	7	UCHL3	1599.2	2	MEGQRWLPLEADPEVTNQFLKQLGLHPNWQF
H8	HLA-B15:27	A/P	0.0350877	3	IGSF3	1248	2	FQRLSPVLYRLTVLQPSPQDTGNYSCHVEEW
H9	HLA-DQA10303-DQB10602	R/W	0.15	5	PCNT	1291.82	1	HLQGVQDGDLEADTEWAARVLGLETEHKVQL

## 4 Discussion

In this study, we conducted neoantigen profiling using an in silico prediction pipeline based only on RNA sequencing and conventional WES/RNA sequencing for breast cancer cell 4T1, lung cancer cell LLC and one breast cancer patient. To conclude, NGS and epitope prediction strategies can identify and prioritise candidate neoantigens using RNA sequencing alone. 107 and 99 candidate neoantigens were eventually obtained by RNA-seq and conventional analysis respectively with forty of the neoantigens were overlapped from 4T1 cells. Four neoantigens were overlapped of 10 mutations with the highest comprehensive score. 559 and 512 candidate neoantigens were eventually obtained by RNA-seq and conventional analysis respectively, 193 neoantigens were overlapped from LLC cells. One neoantigen was overlapped of 10 mutations with the highest comprehensive score. 6/8 of the RNA-derived neoantigens induced significant neoantigen-specific T responses and 3/9 of the conventional neoantigens peptides induced significant immune responses in the 4T1 model. 5/7 of the RNA-derived neoantigens induced significant neoantigen-specific T responses and 4/7 of the conventional neoantigens peptides induced significant immune responses in the LLC model. Hence, the RNA-derived neoantigen prediction obtained more immunogenic mutations compared with conventional analysis. The neoantigen vaccine can elicit an antitumor reaction against mouse breast cancer and lung cancer. The study showed that neoantigen prediction using RNA sequencing alone holds promise as a novel immunotherapeutic approach for treating cancer patients.

The methodology we have developed enables the identification of neoantigens solely through RNA sequencing of a single tumor tissue. This approach concurrently provides genetic mutation information, HLA details, and gene expression data, serving as a comprehensive resource for neoantigen identification. By analyzing the binding affinity between mutated peptides and HLA, coupled with gene expression information, we achieve neoantigen prediction. Furthermore, we have validated the immunogenicity of these predicted neoantigens using both mouse and human samples. The results demonstrate that neoantigens identified solely through RNA sequencing possess immunogenicity and can inhibit tumor growth in mice.

Recent trials of neoantigen vaccines have primarily focused on high TMB tumors (e.g. melanoma) to ensure the identification of a sufficient number of targeted neoantigens (27, 28). In comparison to melanoma, breast cancer typically exhibits much lower TMB values. Limited studies have demonstrated the ability to identify a sufficient number of neoantigens in breast cancer, showing positive effects in postoperative recurrence prevention and combination therapy with conventional treatments (29, 30). Collectively, these preliminary studies suggest that personalized neoantigen vaccines may be feasible even in low-TMB tumors like breast cancer, though their clinical efficacy requires rigorous validation in larger cohorts. The observed immunogenicity of RNA-predicted neoantigens (particularly those from non-canonical regions) warrants further investigation into whether they could complement current immunotherapies in these challenging tumor types.

Our findings align with and extend the seminal work by Laumont et al. (Nat Commun 2016) (31), who demonstrated through proteogenomic analysis that ~10% of MHC-I peptides originate from non-canonical reading frames, including non-coding regions and out-of-frame translations. While their study established the biological reality of such ‘cryptic peptides’ through mass spectrometry validation, our RNA-seq approach provides a complementary strategy to predict these neoantigens computationally before proteomic confirmation. Notably, Laumont’s findings that cryptic peptides exhibit distinct HLA-binding preferences and higher polymorphism rates may explain why our RNA-derived neoantigens showed superior immunogenicity.

Addressing how to expand neoantigen targets in low TMB tumors is particularly necessary. Traditional whole-exome sequencing analyzes gene mutations and only provides information on SNVs and indels in coding gene regions. In addition to coding region SNVs and indels, RNA sequencing can analyze RNA editing, gene fusions, and importantly, variations in non-coding gene regions (32). Our results show that RNA sequencing yields more gene variations than whole-exome sequencing. Previous studies have found a substantial presence of neoantigens in non-coding regions (31, 33). Therefore, RNA sequencing can discover gene variations that whole-exome sequencing cannot. This significantly expands the range of neoantigen acquisition, which is crucial for neoantigen-based immunotherapy in tumors with low TMB, such as breast cancer.

Few tools such as ASNEO, INTEGRATE-neo, and ScanNeo pipelines have been designed to predict neoepitopes from RNA-Seq (15–17). Previous studies collectively demonstrate the utility of these computational pipelines for identifying RNA-derived neoantigens and correlating them with immunotherapy response or survival in observational patient cohorts. While these approaches successfully establish bioinformatic frameworks and reveal clinical associations, validation remains confined to computational predictions and retrospective clinical correlations, lacking direct experimental evidence of neoantigen immunogenicity or therapeutic efficacy. Our study advances this field by providing robust functional validation. We deliver direct experimental proof through 1) vaccinated tumor-bearing mice models demonstrating *in vivo* immunogenicity and tumor protection, and 2) human vaccine efficacy data confirming antigen-specific T-cell responses and clinical benefit. This bridges the gap between computational prediction and therapeutic application, offering tangible evidence for the translational potential of our neoantigen pipeline.

While our study demonstrates the feasibility of RNA-seq-only neoantigen prediction, several limitations must be acknowledged. Most critically, the clinical validation was restricted to a single breast cancer patient, which precludes broad generalizations about the pipeline’s applicability across diverse tumor types or molecular subtypes. This constraint stems primarily from the exploratory nature of this proof-of-concept study, designed to establish technical feasibility rather than clinical efficacy. Further clinical sample verification would be needed to validate the clinical utility of this approach. Although our ELISpot assays demonstrated preliminary evidence of neoantigen-specific IFN-γ responses, we acknowledge that additional functional validations, such as cytotoxicity assays (e.g., T-cell killing or cytokine release), CD8^+^ T-cell expansion analyses, and tetramer staining, would further substantiate the translational potential of these findings. In the current study, human validation was restricted to IFN-γ ELISpot assays using PBMCs; thus, complementary approaches are warranted to comprehensively evaluate the functional immunogenicity of predicted neoantigens. Furthermore, due to the limitation of depth, error rate in sequencing and other technical factors, the identification of gene mutations and the accurate analysis of mutation frequency need to be further improved (34). Finally, there are many technical challenges in peptide synthesis such as sequence complexity (e.g., hydrophobic regions leading to M6/M8 failures), aggregation, storage and handling.

While the current study focused on establishing the immunogenicity of RNA-derived neoantigens, demonstrating antigen specificity through cross-reactivity testing would further strengthen the findings. In future work, we plan to perform additional *in vitro* experiments to test whether splenocytes from immunized mice respond specifically to their target peptides without cross-reacting with unrelated peptides. MHC-matched control tumor models will also be included to distinguish antigen-specific from non-specific immune effects.

## 5 Conclusion

In conclusion, this study indicates the successful identification of new antigens through standalone RNA sequencing. Simultaneously obtaining gene variation information, HLA information, and gene expression information completes the prediction of neoantigens. Initial validation of their potential in antitumor activity has been achieved. Standalone RNA sequencing as a potential improvement for predicting neoantigens, with the prospect of reducing costs and expanding the scope of neoantigen acquisition, is expected to become an essential component of new immunotherapies. However, more research and clinical experiments are needed to verify its feasibility.

## Data Availability

The datasets presented in this study can be found in online repositories. The names of the repository/repositories and accession number(s) can be found in the article/**Supplementary Material**.
