# Supplementary material for: RNA sequencing enables neoantigen discovery and vaccine validation in breast and lung cancer
Source: Front Immunol. 2025 Oct 8;16:1682312. doi: 10.3389/fimmu.2025.1682312 (PMC12540381; doi:10.3389/fimmu.2025.1682312)
Supplement: Supplementary file 1 [file Supplementaryfile1.zip › Supplementary_Material/Supplementary_Material.docx]

Supplementary Material

# Supplementary Figures

**
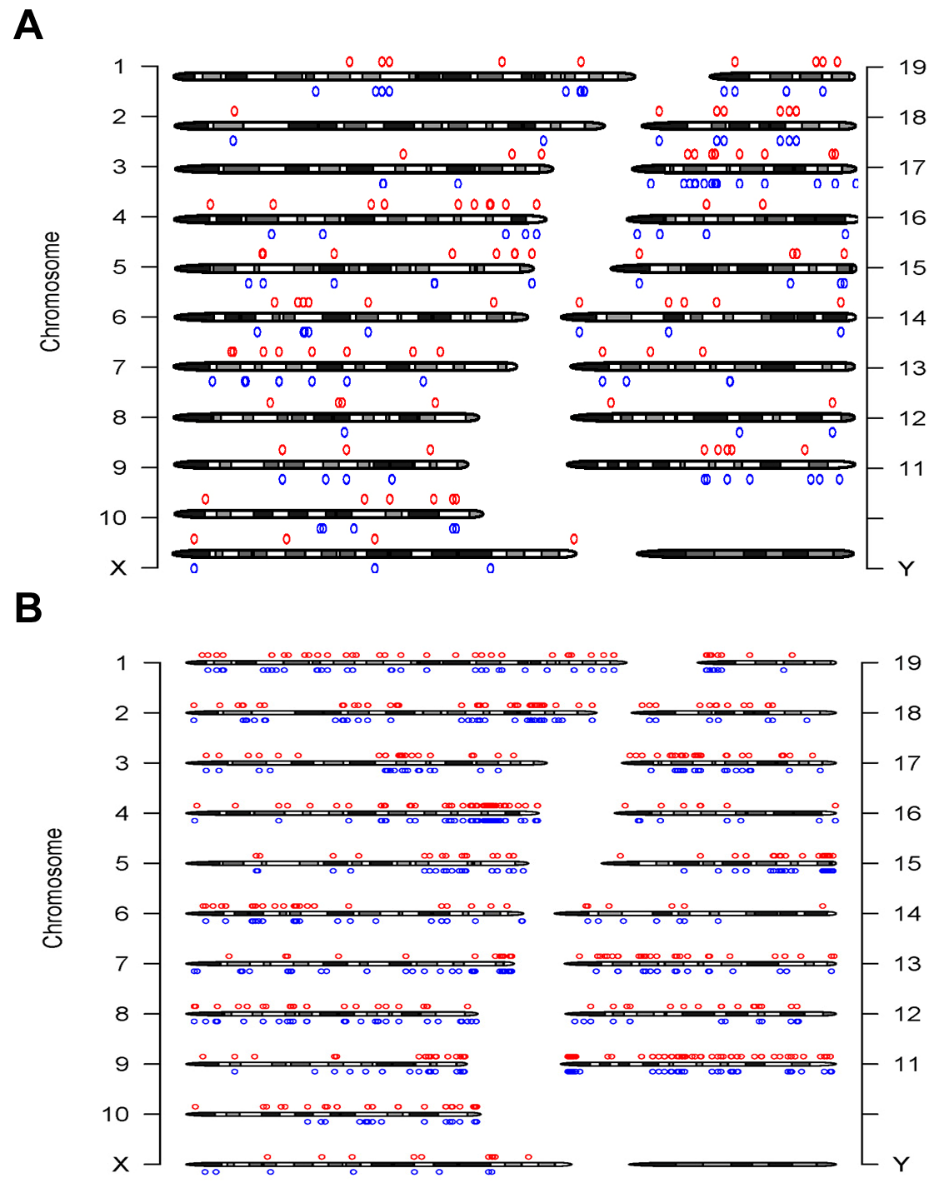
**

**Supplementary Figure 1.** Chromosomal distribution of conventional and RNA-derived neoantigens in breast cancer patients and LLC cells. (A) The chromosomal positions of the neoantigens in breast cancer patient. Red, conventional neoantigens; Blue, RNA-derived neoantigens. (B) The chromosomal positions of the neoantigens in LLC cells. Red, conventional neoantigens; Blue, RNA-derived neoantigens.

**
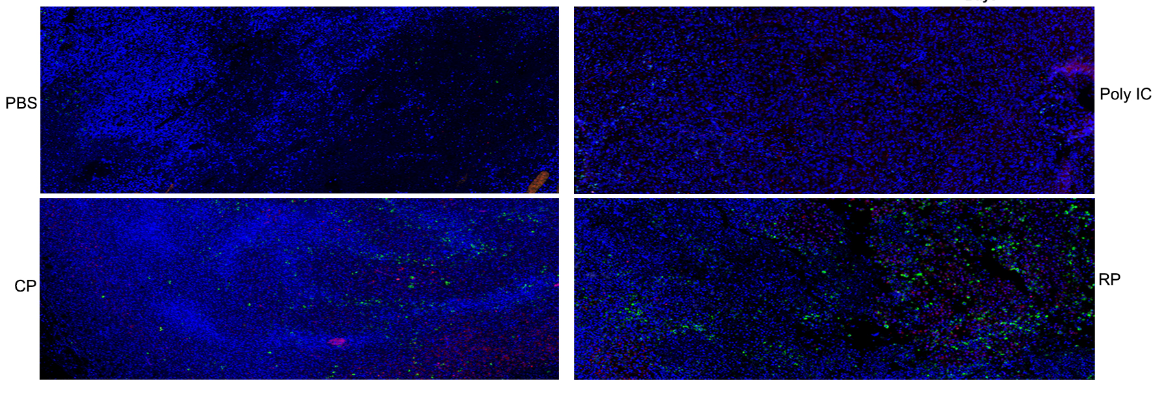
**

**Supplementary Figure 2.** Immunofluorescence (IF) Analysis of tumor tissues. CD3 (orange), CD137 (green).

**
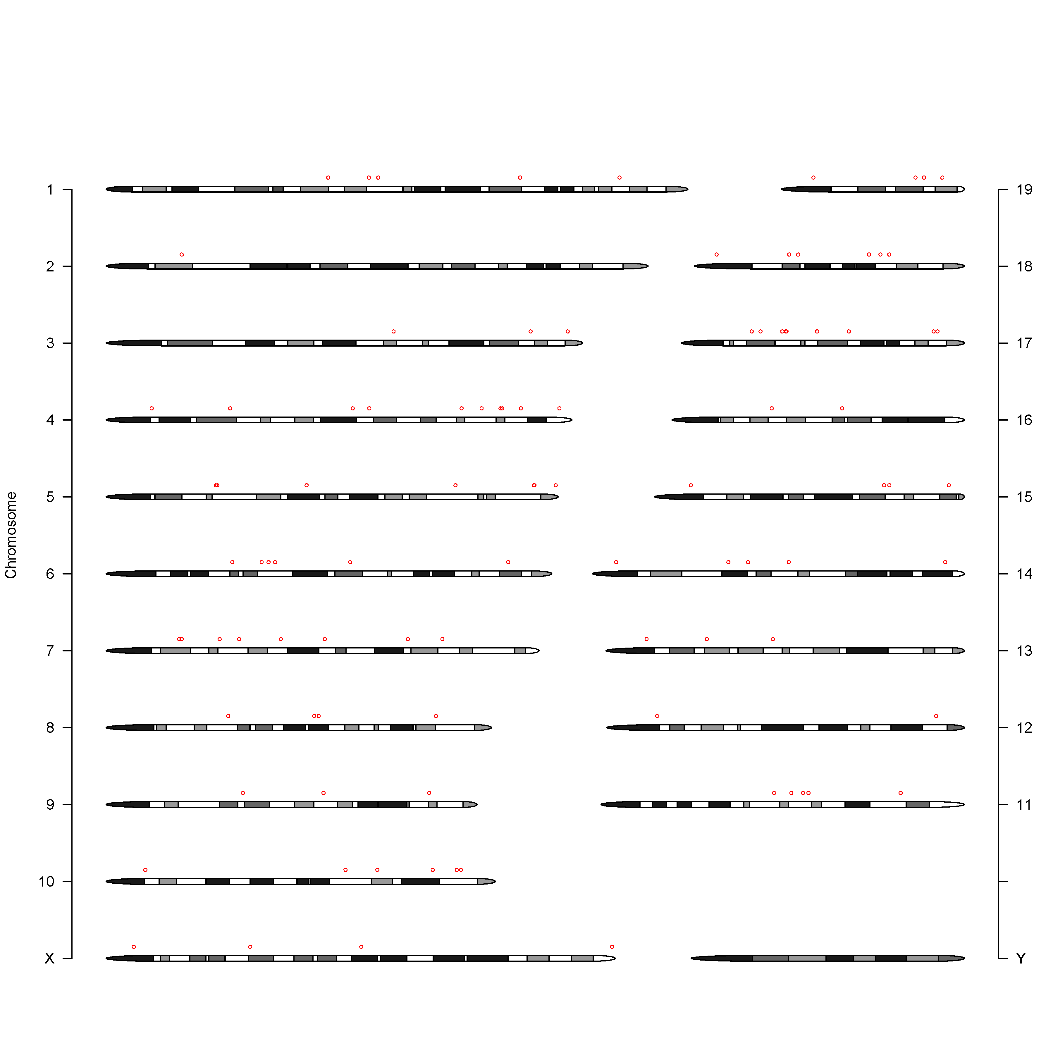
**

**Supplementary Figure 3.** The chromosomal positions of conventional neoantigens in 4T1 cells.

**
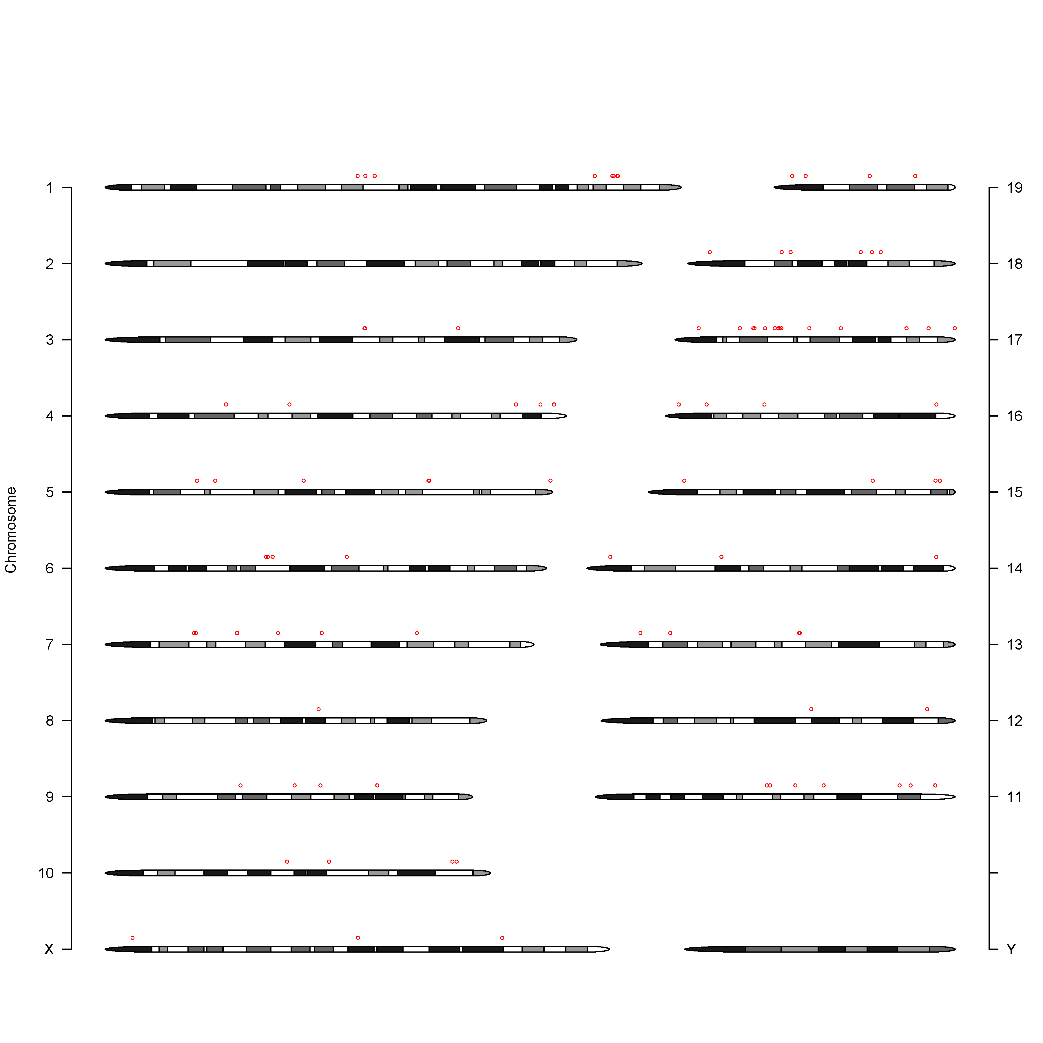
**

**Supplementary Figure 4.** The chromosomal positions of RNA-derived neoantigens in 4T1 cells.
