# Supplementary figures and images for: RNA sequencing enables neoantigen discovery and vaccine validation in breast and lung cancer
Source: Front Immunol. 2025 Oct 8;16:1682312. doi: 10.3389/fimmu.2025.1682312 (PMC12540381; doi:10.3389/fimmu.2025.1682312)

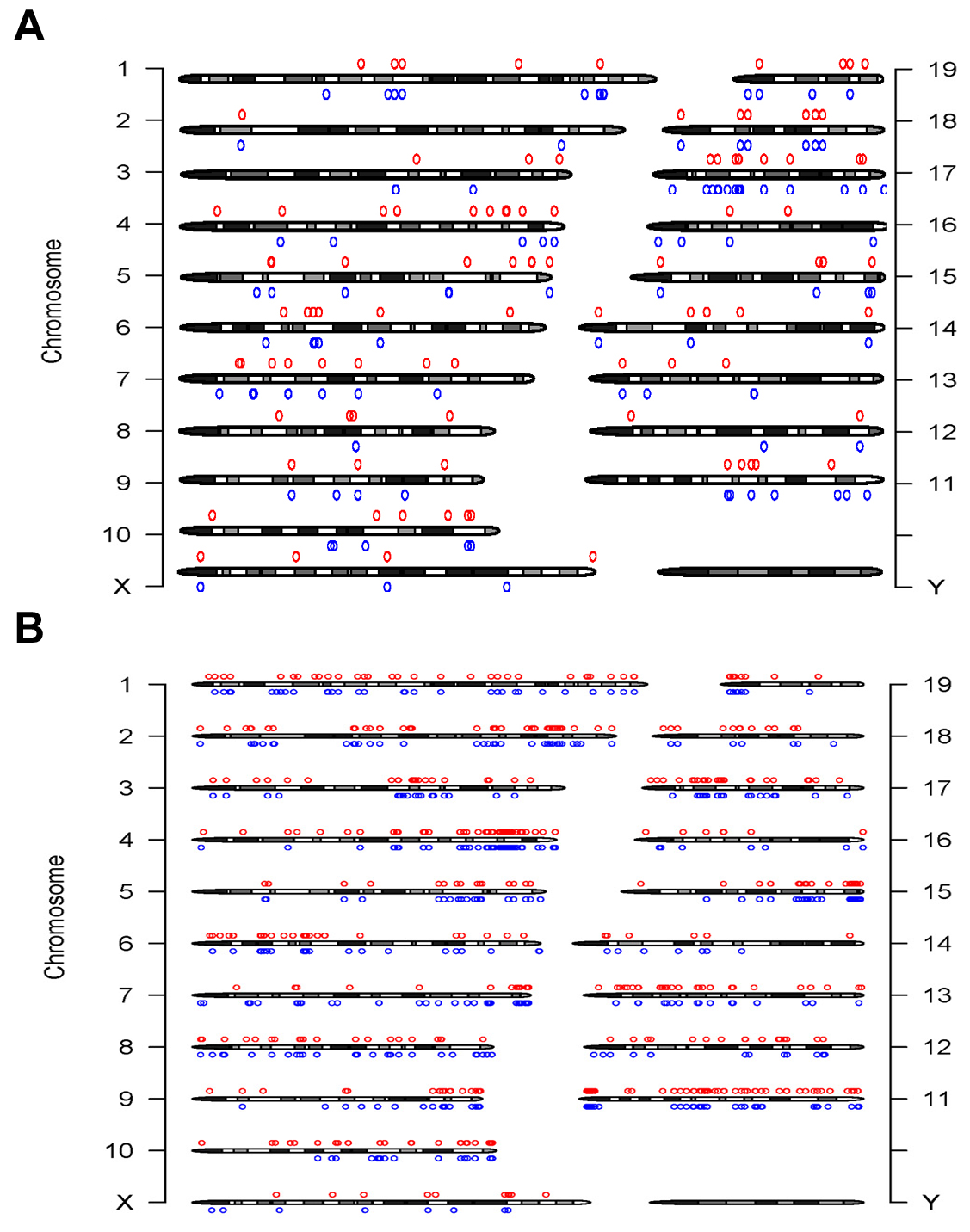

Supplement: Supplementary file 1 [file Supplementaryfile1.zip › Supplementary_Material/Figure S1.tif]

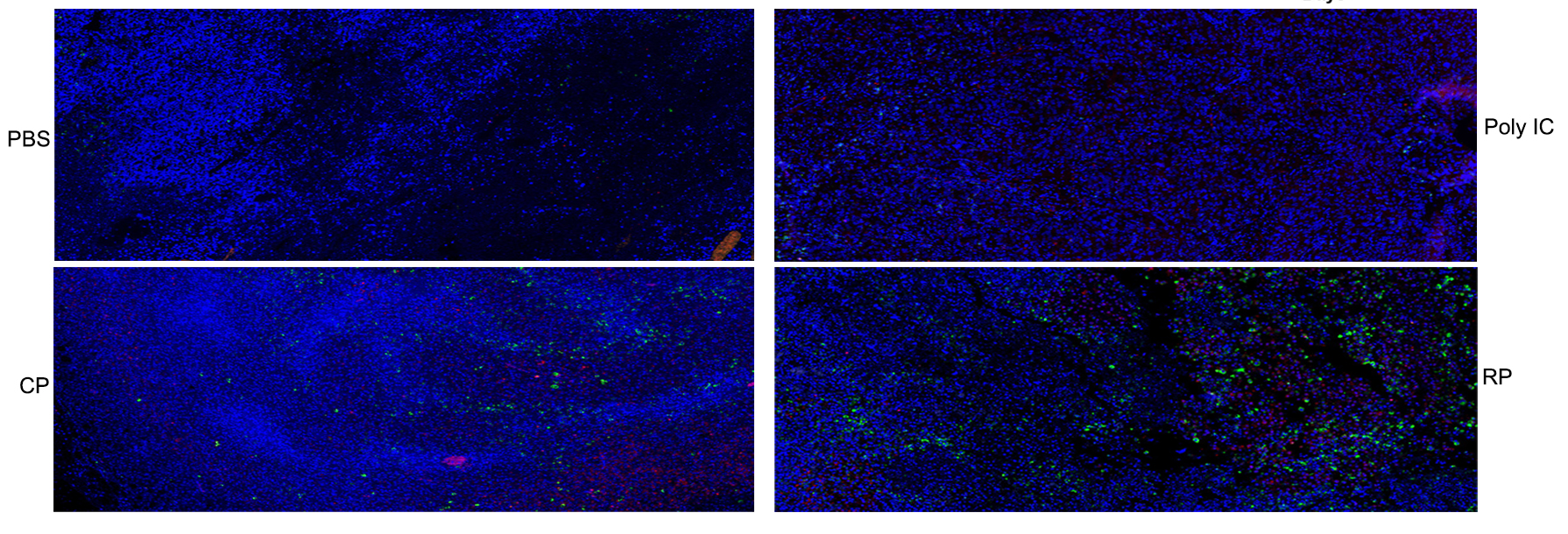

Supplement: Supplementary file 1 [file Supplementaryfile1.zip › Supplementary_Material/Figure S2.tif]

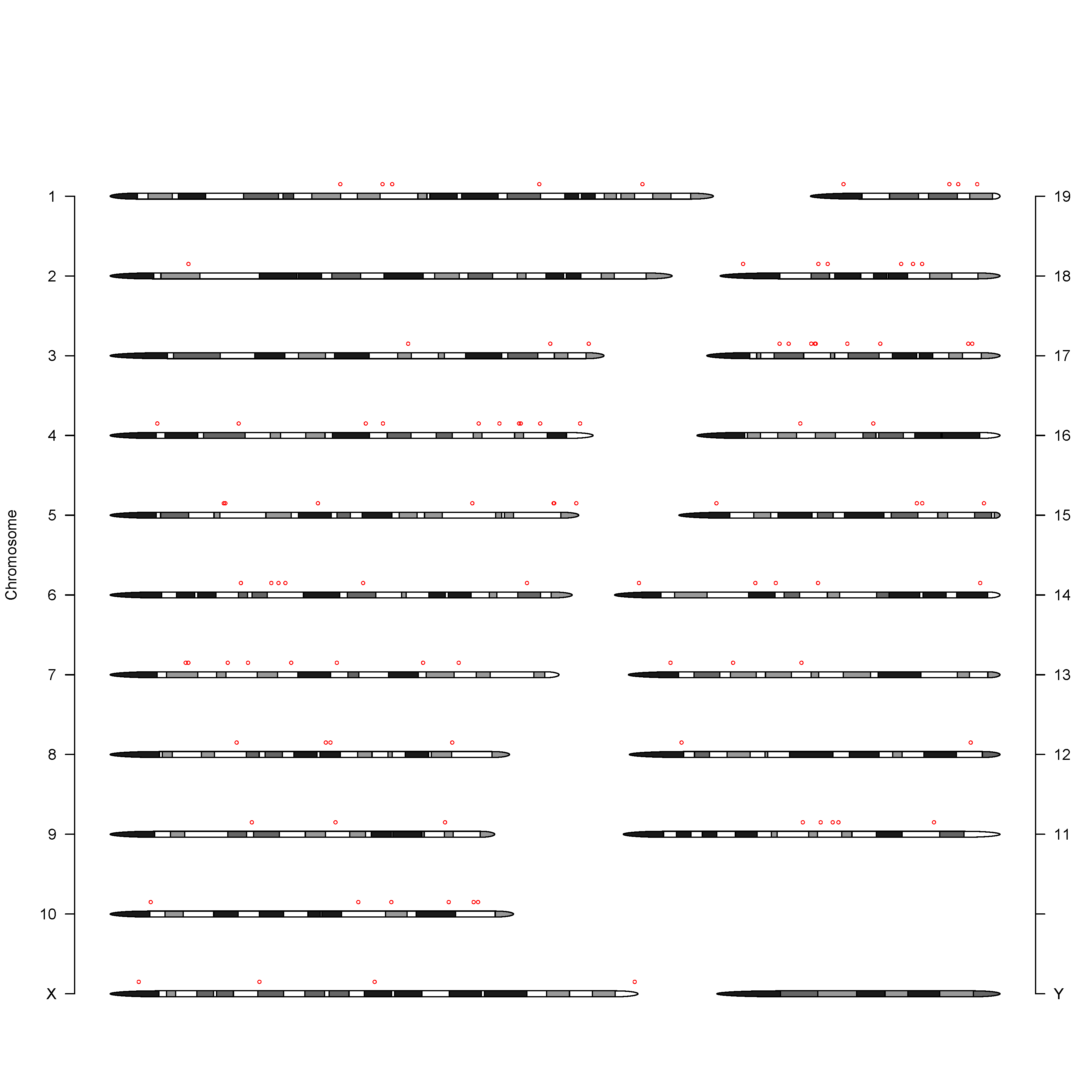

Supplement: Supplementary file 1 [file Supplementaryfile1.zip › Supplementary_Material/Figure S3.png]

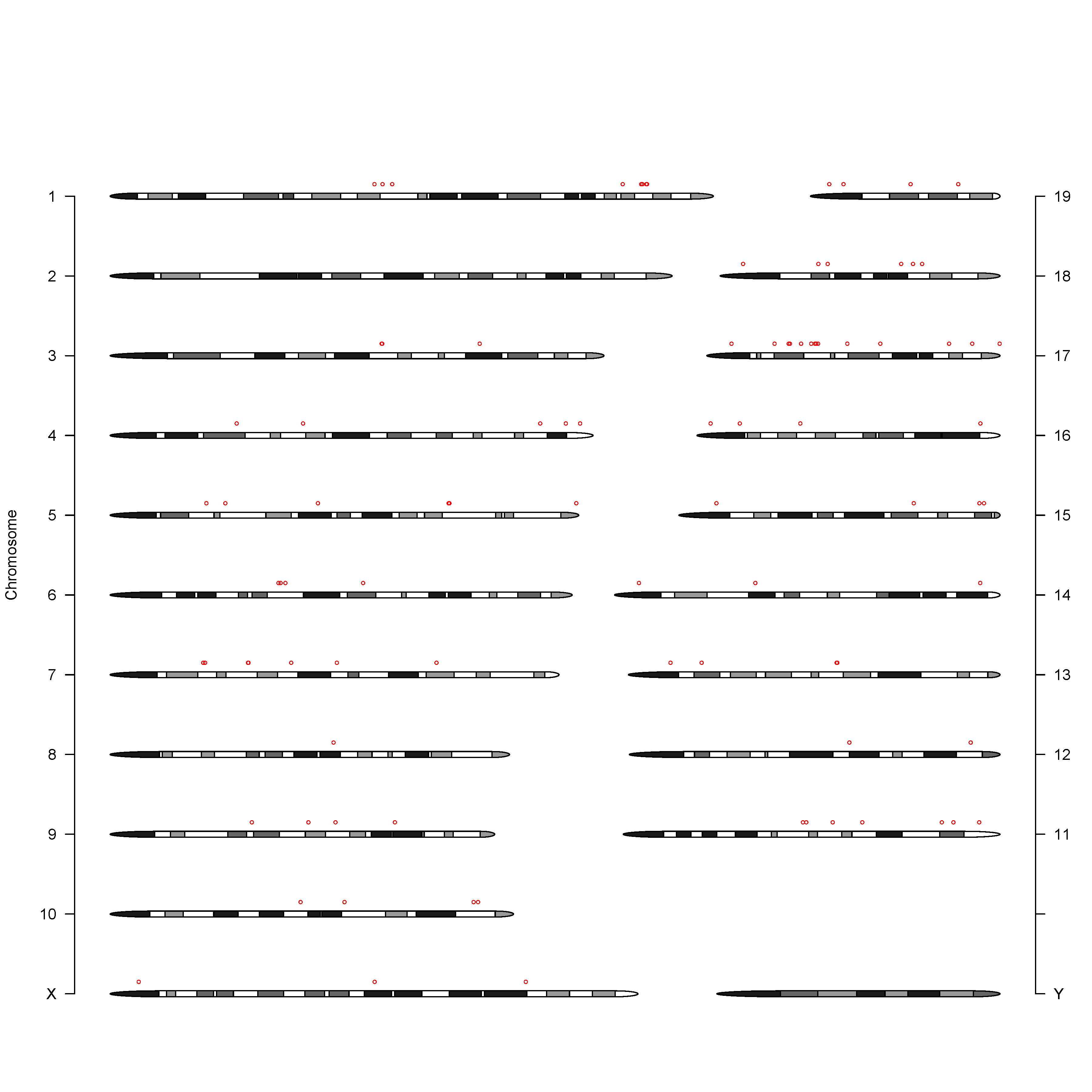

Supplement: Supplementary file 1 [file Supplementaryfile1.zip › Supplementary_Material/Figure S4.png]
